# Double-layer repair of a tuberculous ascending aortic aneurysm: a case report and literature review

**DOI:** 10.1097/RC9.0000000000000366

**Published:** 2026-03-26

**Authors:** Chuong Pham Tran Viet, Khang Cao Dang, Thang Huynh Huu, Thang Ho Duc, Truong Nguyen Hung, Dinh Nguyen Hoang

**Affiliations:** a Department of Cardiovascular Surgery, University Medical Center Ho Chi Minh City, Ho Chi Minh City, Vietnam; b Department of Cardiovascular and Thoracic Surgery, Faculty of Medicine, University of Medicine and Pharmacy, Ho Chi Minh City, Vietnam

## Abstract

**Introduction and Importance:**

Tuberculous aneurysm of the ascending aorta is extremely rare and life-threatening, particularly in elderly patients. Early recognition and appropriate surgical management are essential to prevent fatal rupture.

**Case Presentation:**

An 84-year-old man presented with hemoptysis and chest tightness. Computed tomography revealed a saccular aneurysm of the ascending aorta with sternal erosion. During surgery, the aneurysm showed a caseous appearance with dense adhesions and infiltration into adjacent tissue. Frozen section demonstrated granulomatous inflammation suggesting tuberculous aortitis, which was later confirmed by postoperative Xpert MTB/RIF assay. A double-layer aortic repair was performed using an inner bovine pericardial patch and an outer Gelweave graft. The patient recovered well under antituberculous therapy and remained stable at 7-month follow-up.

**Clinical Discussion:**

Tuberculous aortitis in elderly patients is challenging due to degenerative aortic wall changes and hypertension. Intraoperative frozen section combined with gross findings played a key role in guiding the surgical strategy. The double-layer repair provided infection control and mechanical durability, consistent with current literature supporting biologic–synthetic hybrid reconstruction in infected aneurysms.

**Conclusion:**

A tuberculous aneurysm of the ascending aorta should be considered in atypical cases presenting with hemoptysis. In elderly patients with degenerative aortic walls and hypertension, double-layer repair offers effective infection control and durability when combined with appropriate antituberculous therapy. Early diagnosis and tailored surgical planning are crucial for favorable outcomes.

## Introduction

Tuberculous aneurysms of the ascending aorta are rare but life-threatening, often presenting atypically without systemic signs[1]. These lesions may mimic other pathologies, such as localized ruptured aneurysms or pulmonary masses on imaging[2]. We report an 84-year-old man presenting with hemoptysis and sternal erosion. Computed tomography revealed a contained aneurysm of the ascending aorta with adjacent perianeurysmal inflammation. Standard surgical management with prosthetic grafts in infected or inflamed fields carries a substantial risk of reinfection and complications. Unlike most previously reported cases, which predominantly involve younger patients or other aortic segments, this high-risk elderly patient was managed using a biologic–synthetic double-layer repair strategy to reinforce the ascending aorta. This case illustrates the diagnostic and therapeutic challenges of managing tuberculous aortitis involving the ascending aorta.

HIGHLIGHTS

- Tuberculous aneurysm of the ascending aorta is rare and life-threatening
- Intraoperative frozen section combined with gross findings suggested mycotic aneurysm and guided management
- Double-layer repair may decrease the risk of reinfection and improve mechanical durability
- Early targeted antituberculous therapy ensured infection control and good recovery

This case report has been reported in line with the SCARE 2025[3].

## Case presentation

An 84-year-old man with a known history of hypertension presented to the emergency department with a 3-week history of intermittent hemoptysis, described as small amounts of bright red blood, accompanied by chest tightness. There was no history of fever, weight loss, or night sweats. Physical examination was unremarkable; he was hemodynamically stable with normal oxygen saturation on room air.

**Figure 1.**
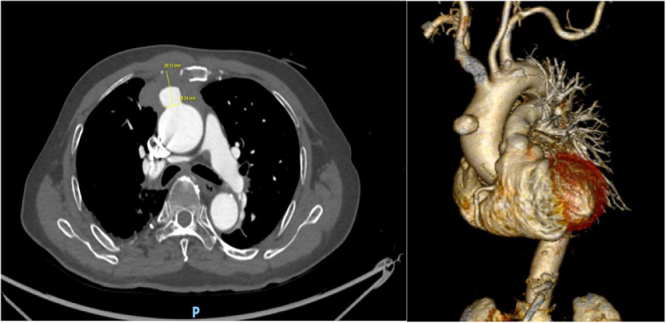
Contrast CT scan showing a focal saccular aneurysm of the anterior wall of the ascending aorta with peripheral enhancement, adjacent periaortic inflammatory changes, and sternal erosion – features suggestive of an infectious aneurysm.

Upon admission, transthoracic echocardiography showed the dilated proximal ascending aorta (maximum diameter of 43.5 mm) with the aortic root measured 44 mm. The aortic and mitral valve was mild regurgitation and a mildly dilated left ventricle with preserved ejection fraction (55%).Computed tomography (CT) demonstrated a saccular aneurysm arising from the anterior wall of the ascending thoracic aorta, measuring approximately 20 × 15 × 18 mm with a 14-mm neck (Fig. 1) . Adjacent sternal bone erosion was noted, raising suspicion for a mycotic aneurysm. Additional findings included scattered non-specific pulmonary nodules, fibrotic and atelectatic changes with consolidation in the right middle lobe (segments S3 and S5), and mildly enlarged mediastinal lymph nodes. Ziehl–Neelsen staining of the sputum smear for *Mycobacterium tuberculosis* was negative. Because of economic constraints, PET-CT was not performed, although it might have provided additional information regarding potential occult infectious foci.

## Technique

**Figure 2.**
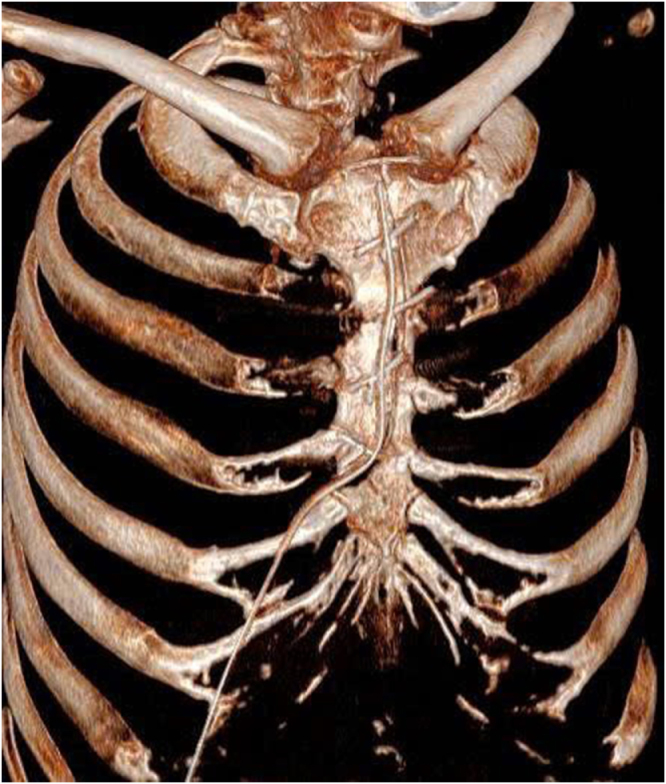
Right-sided J-shaped sternotomy at the fourth intercostal space to minimize risk of aneurysmal injury.

**Figure 3.**
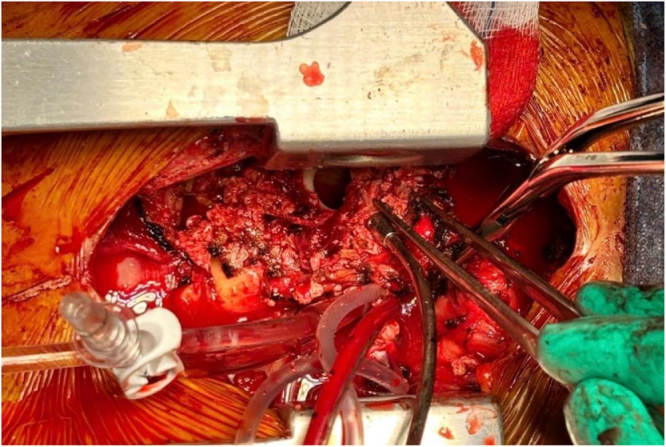
Intraoperative view of the aneurysm showing caseous necrosis with dense adhesions and surrounding tissue invasion.

**Figure 4.**
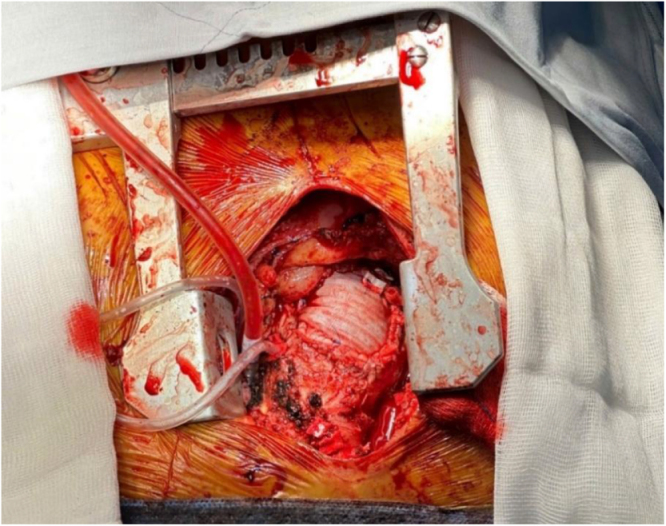
The double-layer aortic repair and reimplantation of the aortic arch branches.

The close proximity and rightward deviation of the aneurysm relative to the sternum posed a significant risk of inadvertent injury to the aneurysmal sac during sternal division. To mitigate this risk, we initiated percutaneous femoral artery and vein cannulation and administered systemic heparin to establish cardiopulmonary bypass (CPB). A right-sided J shaped sternotomy was performed at the level of the fourth intercostal space to avoid direct trauma to the aneurysm (Fig. 2). The aneurysm exhibited a caseous appearance with dense perianeurysmal adhesions and infiltration into adjacent tissues (Fig. 3). Intraoperative frozen section was performed to exclude malignancy. Histology revealed chronic granulomatous inflammation, highly suggestive of tuberculous aortitis, although other infectious etiologies could not be excluded. Based on these findings, the ascending aorta near the brachiocephalic trunk was exposed for aortic cross-clamping. Proximal dissection was limited to just above the sinotubular junction to allow antegrade cardioplegia, avoiding manipulation close to the aneurysm. Following initiation of full cardiopulmonary bypass, systemic cooling was achieved to 32°C prior to opening the aneurysm. The aneurysm was resected extensively to visually normal tissue. The aorta was then reconstructed using a double-layer technique, with an inner bovine pericardial patch (Edwards bovine pericardial patch) chosen for its resistance to infection, and an outer graft (Gelweave TM Straight) to provide better pressure support. After completion of the double-layer aortic repair (Fig. 4), we initiated weaning from cardiopulmonary bypass, placed drainage tubes at the surgical site, and closed the chest. Cardiopulmonary bypass time was 92 minutes, and crossclamp time was 52 minutes. Following hospital discharge on postoperative day 10, the patient was initiated on a 6-month treatment regimen consisting of ethambutol, rifampicin, pyridoxine, and isoniazid, after Mycobacterium tuberculosis was detected by Xpert MTB/RIF assay. Seven months after the operation, the patient was in good condition without any complications. He had no fever, and all hematological parameters were normal.

## Discussion

Infected aneurysm of the ascending aorta is rare but highly lethal, so early diagnosis is critical; however it is challenging due to often presenting with nonspecific symptoms and atypical imaging features. The diagnosis of tuberculous aneurysm often relies on culture, histology, intraoperative findings, or prior tuberculosis history[2]. The patient in our case had no documented history of pulmonary tuberculosis or prior antituberculous therapy. Ascending aortic aneurysms may arise through direct extension, vasa vasorum spread, or intimal seeding, as described by Long *et al*, and our patient’s presentation is consistent with these mechanisms[1].

From a diagnostic perspective, differential diagnoses for a saccular ascending aortic lesion presenting with hemoptysis include contained ruptured aneurysm, mediastinal malignancy, and infectious (mycotic) aneurysm. In our case, malignancy was initially considered because of adjacent sternal erosion; however, the absence of a solid mass on computed tomography and exclusion of neoplasia on intraoperative frozen section made this diagnosis less likely.

Contrast CT remains a key diagnostic tool, especially where advanced imaging is unavailable. As described by Xing *et al*, tuberculous aortic aneurysms typically present as focal saccular or pseudoaneurysmal dilatations with irregular walls, peripheral enhancement, periaortic inflammatory changes, and erosion of adjacent structures[2]. The CT findings in our patient closely matched these features, supporting an infectious etiology and prompting early surgical intervention.

In such scenarios, intraoperative frozen section can provide valuable evidence to guide management decisions. In this case, the frozen section revealed chronic granulomatous inflammation, and together with the gross appearance of the lesion suggestive of caseous necrosis, it raised suspicion for tuberculous infection of the aortic wall. Based on this, we considered the possibility of an infected (mycotic) aneurysm of the ascending aorta, which allowed us to adopt a safer and more appropriate treatment strategy.

In our case, we adopted a double-layer reconstruction technique to balance infection control and mechanical durability. The inner layer consisted of a bovine pericardial patch, selected for its favorable biocompatibility and its reported performance in contaminated surgical fields. While some studies and meta-analyses have suggested that biologic materials may offer a lower reinfection risk compared to synthetic grafts in the context of mycotic aneurysms or infected aortic grafts, these findings remain largely observational and theoretical^[^4,5^]^. To further mitigate the risk of reinfection, we initiated antituberculous therapy postoperatively following confirmation of *Mycobacterium tuberculosis* via Xpert MTB/RIF assay, ensuring systemic infection control in parallel with surgical management.

However, biologic patches alone may have limited tensile strength in the setting of systemic arterial pressure, particularly in elderly patients with reduced vascular elasticity and increased hemodynamic fragility. Recco *et al* demonstrated that autologous pericardium exhibited poor mechanical performance under pulsatile flow, raising concerns about early graft failure when used without external support[6]. Given our patient’s advanced age and the anatomical location of the lesion (ascending aorta), we opted to reinforce the reconstruction with an outer synthetic graft to enhance structural durability and reduce the risk of early mechanical complications.

Most previously reported cases (Table 1) involved total graft replacement or stent-graft repair, with minimal use of biologic reinforcement. Our strategy preserved healthy aortic segments and applied a double-layer repair—bovine pericardial patch for infection resistance, reinforced externally with a synthetic graft for strength—combined with postoperative antituberculous therapy, providing a less invasive alternative with the potential to further reduce reinfection risk.

**Table 1:** Case reports of tuberculous aortic aneurysm.

Case (year) [Citation]	Age/Sex	Presentation & diagnosis	Surgical repair	Antituberculous therapy	Outcome & follow-up	Special notes
Pathirana *et al*, (2015)[7]	40/F	Severe aortic regurgitation, 54-mm ascending aneurysm; histopathology confirmed	Aortic valve replacement + Albo graft root replacement	RHZE × 2 months + RH × 10 months continuation	Uneventful recovery; good graft function	Use of biological graft (Albo graft) for lowering infection risk
Syed Shahabuddin *et al*, (2013)[8]	25/M	Chest pain, dyspnea, recurrent hemoptysis; large pseudoaneurysm of the ascending aorta confirmed on CT, histology tuberculosis	Excision + in situ 22-mm Dacron graft	Postop ATT started based on histological confirmation	Smooth recovery; transient heart block	Prosthetic graft used with standard ATT
Gopalan *et al*, (2015)[9]	31/F	Fever, weight loss, new mediastinal widening; large ascending aortic pseudoaneurysm on CT; confirmed by biopsy	Repair with ascending aorta patch using collagen-coated polyester pericardial patch	ATT continued for 9 months	Uneventful recovery; doing well at 1-year follow-up	Rare ascending aortic pseudoaneurysm in disseminated TB; patch repair with ATT

ATT, antituberculous therapy; CT, computed tomography; F, female; M, male; RH, rifampin (R) and isoniazid (H); RHZE, standard four‑drug antituberculous regimen consisting of rifampin (R), isoniazid (H), pyrazinamide (Z), ethambutol (E); TB, tuberculosis.

## Conclusion

Infection should be considered for atypical ascending aortic lesions, with intraoperative histology guiding management. Targeted debridement with clamping on uninvolved aorta may allow safer control than EVAR in suspected infection. A combined biologic–synthetic “double-layer” repair, paired with early, Xpert-guided antituberculous therapy, is intended to balance compatibility and mechanical durability, and may help lower reinfection risk compared with full prosthetic replacement in high-risk settings.

## Data Availability

All of the material is available and owned by the authors and/or no permissions are required.
